# The Temperature Dependence of Phytoplankton Stoichiometry: Investigating the Roles of Species Sorting and Local Adaptation

**DOI:** 10.3389/fmicb.2017.02003

**Published:** 2017-10-23

**Authors:** Gabriel Yvon-Durocher, Charlotte-Elisa Schaum, Mark Trimmer

**Affiliations:** ^1^Environment and Sustainability Institute, University of Exeter, Penryn, United Kingdom; ^2^School of Biological and Chemical Sciences, Queen Mary University of London, London, United Kingdom

**Keywords:** global warming, phytoplankton, stoichiometry, rapid evolution, species sorting

## Abstract

The elemental composition of phytoplankton (C:N:P stoichiometry) is a critical factor regulating nutrient cycling, primary production and energy transfer through planktonic food webs. Our understanding of the multiple direct and indirect mechanisms through which temperature controls phytoplankton stoichiometry is however incomplete, increasing uncertainty in the impacts of global warming on the biogeochemical functioning of aquatic ecosystems. Here, we use a decade-long warming experiment in outdoor freshwater ponds to investigate how temperature-driven turnover in species composition and shifts in stoichiometric traits within species through local thermal adaptation contribute to the effects of warming on seston stoichiometry. We found that experimental warming increased seston C:P and N:P ratios, while the C:N ratio was unaffected by warming. Temperature was also the dominant driver of seasonal variation in seston stoichiometry, correlating positively with both C:P and N:P ratios. The taxonomic composition of the phytoplankton community differed substantially between the warmed and ambient treatments indicating that warming resulted in differential sorting of species from the regional pool. Furthermore, taxonomic composition also changed markedly over the year within each of the warmed and ambient treatments, highlighting substantial temporal turnover in species. To investigate whether local adaptation also played an important role in shaping the effects of warming on seston stoichiometry, we isolated multiple strains of the cosmopolitan alga, *Chlamydomonas reinhardtii* from across the warmed and ambient mesocosms. We found that warmed isolates had higher C:P and N:P ratios, shifts that were comparable in direction and magnitude to the effects of warming on seston stoichiometry. Our results suggest that both species sorting and local adaptation are likely to play important roles in shaping the effects of warming on bulk phytoplankton stoichiometry and indicate that major shifts in aquatic biogeochemistry should be expected in a warmer world.

## Introduction

The stoichiometry of carbon (C), nitrogen (N), and phosphorous (P) in phytoplankton biomass set important constraints on the biogeochemistry of aquatic ecosystems, shaping patterns of nutrient limitation (Elser et al., 2009; Bonachela et al., 2013; Alexander et al., 2015), recycling (Sterner and Elser, 2002), material transfer, and C sequestration in planktonic ecosystems (Galbraith and Martiny, 2015). Until recently it was assumed that the ratios of these elements were maintained in relatively fixed proportions (i.e., the Redfield ratio, C:N:P = 106:16:1) and exhibit tight coupling between organic and inorganic pools (Geider and La Roche, 2002). It is now widely recognized that the C:N:P stoichiometry of phytoplankton is highly variable across multiple spatial, temporal and organizational scales (Geider and La Roche, 2002). Such variation has been linked to directional changes in abiotic factors (e.g., light, CO_2_ and nutrients), with temperature often cited as a key determinant of phytoplankton stoichiometry (Woods et al., 2003; Hessen, 2005; Martiny et al., 2013; Toseland et al., 2013; Yvon-Durocher et al., 2015b). However, despite much recent progress, we still lack a detailed understanding of the multiple direct and indirect mechanisms through which temperature controls phytoplankton stoichiometry across scales of time, space and biological organization, limiting our ability to forecast impacts of global warming on macronutrient cycles.

The elemental stoichiometry of a phytoplankton cell is the result of resource allocation to different subcellular constituents that vary in their C, N, and P content and together determine the cell's macromolecular composition (Shuter, 1979; Daines et al., 2014). For example, polysaccharides, lipids and carbohydrates are major sinks for C allocation; proteins represent the major fraction of the cell's investment of N, while ribosomal RNA and phospholipids account for a large part of P allocation (Geider and La Roche, 2002). Temperature is a key driver phytoplankton metabolism (Raven and Geider, 1988; Thomas et al., 2012; Sal et al., 2015; Padfield et al., 2016) and recent work suggests that changes in temperature alter the cell's optimal allocation to C, N and P pools via phenotypic plasticity (i.e., acclimation; Toseland et al., 2013; Daines et al., 2014). The “*temperature-dependent physiology*” hypothesis predicts that organisms growing at higher temperatures should have higher N:P ratios because they require fewer P-rich ribosomes, relative to N-rich proteins, to sustain growth and maintenance (Woods et al., 2003; Toseland et al., 2013; Yvon-Durocher et al., 2015b). In phytoplankton, such a shift could occur if the rates of photosynthesis by N-rich photosynthetic proteins exhibit weaker temperature dependence than protein synthesis by P-rich ribosomes (Yvon-Durocher et al., 2015b). A recent meta-analysis of temperature manipulation experiments on 9 species of marine and freshwater phytoplankton demonstrated a positive association between temperature and C:P and N:P ratios, but not C:N, suggesting that, in support of the “*temperature-dependent physiology*” hypothesis, changes in cellular stoichiometry were attributable to declines in P content as cells acclimate to warmer temperatures (Yvon-Durocher et al., 2015b). Furthermore, direct measurements of cellular allocation to RNA in chlorophytes and diatoms have also revealed rapid declines as populations acclimate to warmer growth temperatures (Toseland et al., 2013; Hessen et al., 2017). Evidence in support of the “*temperature-dependent physiology*” hypothesis is not unequivocal however, with acclimation experiments on the marine cyanobacterium *Prochlorococcus* revealing that increases in C:P and N:P at higher temperatures were driven by elevated C and N contents, rather than declines in P, in warm acclimated cells (Martiny et al., 2016a). Nevertheless, the current weight of evidence indicates that physiological plasticity in response to rapid changes in temperature can cause substantial shifts in algal stoichiometry within species that are at least as large as those observed across species, irrespective of the underlying molecular and biochemical mechanisms.

Phytoplankton stoichiometry exhibits substantial variation among the major lineages, presumably reflecting their divergent evolutionary histories (Quigg et al., 2003; Litchman et al., 2007; Litchman and Klausmeier, 2008; Finkel et al., 2009). Red lineage algae, which include the diatoms and coccolithophores and dominate the eukaryotic contribution to contemporary global marine primary production, have relatively low N:P ratios (Quigg et al., 2003). In contrast, green lineage algae, which include the chlorophytes and prasinophytes that dominated ocean productivity in the Proterozoic and Paleozoic, often have relatively high N:P ratios (Quigg et al., 2003). Thus, owing to the substantial differences in stoichiometric traits that exist among phytoplankton taxa, environmental filtering of species along thermal gradients has the potential to drive variation in the bulk stoichiometry of seston when species-level selection is systematic with respect to temperature and elemental stoichiometry (Martiny et al., 2016b).

Rapid evolutionary responses to directional environmental change could also play an important role in shaping the effects of warming on phytoplankton stoichiometry and biogeochemical macronutrient cycling. Indeed, recent work experimentally evolving both marine and freshwater phytoplankton under future temperature and CO_2_ scenarios have shown that adaptation can be rapid (<1 year or a few hundred phytoplankton generations) and often involves changes in stoichiometric traits (e.g., C:N; Schlueter et al., 2014; Schaum et al., 2016). However, experiments explicitly investigating rapid evolutionary shifts in phytoplankton C:N:P stoichiometry in response to warming are currently lacking. We therefore have limited understanding of the direction, magnitude and tempo over which stoichiometric traits might evolve as phytoplankton adapt to warming and consequently, the contribution of rapid evolution to changes in biogeochemical cycles.

Here, we use a decade-long warming experiment in outdoor freshwater ponds to investigate the interplay between species sorting and rapid evolution in shaping the effects of temperature on phytoplankton stoichiometry. We first present a detailed analysis of both seasonal changes in phytoplankton stoichiometry within ponds as well as the long-term differences between treatments attributable to experimental warming. We then assess the role of local adaptation by quantifying changes in C:N:P stoichometry in strains of the cosmopolitan alga, *Chlamydomonas reinhardtii*, isolated from both our ambient and warmed ponds. We have previously shown that *C. reinhardtii* strains isolated from the warmed and ambient treatments are locally adapted to the different thermal regimes imposed by experimental warming and exhibit fitness trade-offs when reciprocally transplanted in the warmed and ambient treatments (Schaum et al., 2017). Based on work in both marine and freshwater systems over latitudinal and temporal (seasonal) thermal gradients, we expect the C:P and N:P ratios of the seston in our experiment to increase with warming and exhibit positive seasonal temperature dependence (Hessen, 2005; Martiny et al., 2013; Yvon-Durocher et al., 2015b). Given the highly dynamic nature of phytoplankton communities, we hypothesize that seasonal variation and treatment effects on bulk stoichiometry will emerge from temperature driven turnover in the taxonomic composition of the algal assemblages. However, we also predict that if temperature driven adjustments in sub-cellular allocation to C, N and P pools that increase C:P and N:P in warmer environments (as expected under the “*temperature-dependent physiology*” hypothesis) also increase fitness, then they will be reinforced through evolutionary adaptation. Consequently, we hypothesize that isolates of the cosmopolitan alga, *C. reinhardtii*, that have adapted to the thermal regimes in the warmed mesocosms will have higher C:P and N:P ratios than their counterparts from the ambient treatments.

## Materials and methods

### Mesocosm experimental design

The mesocosm facility was established in 2005 and consists of 20 artificial ponds of ~1 m^3^ volume, 50 cm depth, sited in southern England (Freshwater Biological Association Rivers Laboratory, East Stoke, 2°10′W, 50°13′N), designed to be broadly representative of mid-latitude shallow lakes (Yvon-Durocher et al., 2010). Warming of 4–5°C above ambient began in half of the ponds in 2006 by maintaining a constant differential between thermocouples in a pair of warmed and ambient ponds. The ponds contain well-established benthic and pelagic communities including assemblages of macrophytes, phytoplankton, algal biofilms, and invertebrates; for a detailed description of the community composition see previous publications from this facility (Yvon-Durocher et al., 2010, 2015a; Dossena et al., 2012). Sediments are comprised of 8–10 cm of fine sands with a developed organic layer of 1–3 cm (**Table 2**).

### Phytoplankton sampling

The plankton community in each of the mesocosms was sampled every 2 months between July 2011 and May 2012 (6 sampling occasions in total). The phytoplankton composition data presented here are reanalyzed from those in Yvon-Durocher et al. (2015a). The entire water column from the sediment surface to the water surface was sampled using a 0.8 m-length tube sampler (Volume: 2 L), which was positioned at random in each mesocosm on each date. Each sample was passed through a 100 μm aperture sieve to remove the zooplankton. A 100 mL sub-sample of <100 μm fraction was preserved in 1% Lugol's iodine for microscope analysis of the phytoplankton community composition, while the remaining material was filtered through a pre-ashed, Whatman GF/F filter (0.7 μm nominal pore size) in duplicate and then immediately frozen at −20°C prior to analysis of particulate nutrients.

### Seston stoichiometry

Filters (GF/F) were dried for 48 h at 60°C. The dry weight of particulate matter on the filter was calculated and used to standardize by the sample mass in further analyses. One of the two filters was acidified (1 M HCl) to remove carbonates and used for the analysis of particulate organic carbon and nitrogen using a Sercon 20-22 IRMS. The other was used for determining particulate organic phosphorous on a segmented flow auto-analyser (Skalar, San++, Breda, Netherlands) following complete oxidation with potassium persulfate.

### Taxonomic characterization of the phytoplankton community

Phytoplankton <100 μm were counted using a LEICA DMIRB inverted microscope at 400x magnification, following the Utermöhl method. The microscope was connected to an interactive image analysis system (LEICA EC3 camera and LAS software) to allow for a higher magnification. For each sample, at least 400 individuals (single cell, colony or filament) were counted, measured and identified. Counts were converted to volumetric estimates of abundance (organisms mL^−1^) based on the volume of sample analyzed, which varied between 1 and 25 mL depending on the density of organisms. In total, 171 taxa were identified, 85% of which were identified to species level; the remaining 15% were identified to genus or class, or were undetermined.

### Measuring gross primary production

Rates of gross primary production (GPP) were measured over a 24 h diel cycle for each replicate mesocosm on the sampling months described above using the free water dissolved oxygen (DO) change technique (Staehr et al., 2010). Measurements of DO and temperature were taken every 15 min for 24 h at mid-depth (0.25 m) in the water column of each pond with YSI 600XLM multi-parameter Sondes, equipped with 6562 rapid pulse™ dissolved oxygen sensors. Light intensity was measured using a Licor spherical quantum sensor (LI-193, Licor USA) positioned at mid-depth (0.5 m) in the water column of a single ambient mesocosm in the center of the pond array. These measurements allow for quantification of seasonal variation in light intensity but do not allow us to quantify potential differences among treatments in light penetration. Light intensity was measured every minute and logged as 15-min averages using a Licor LI-1500 data logger. Prior to deployment, the multi-parameter Sondes were calibrated in water-saturated air with a correction for barometric pressure. Calibration accuracy was verified by monitoring the DO concentration of water-saturated air for 10 min and checking against 100% O_2_ saturation for the measured temperature and pressure. Measurements of DO, wind speed at 1.7 m (Cole-Parmer, WS-821), and light intensity at mid-depth in the water column were used to calculate GPP and R_eco_ following the methods outlined in Staehr et al. (2010).

### Isolation and characterization of *Chlamydomonas reinhardtii*

We isolated *Chlamydomonas*, a known cosmopolitan and highly abundant genus in the heated and ambient mesocosms (Yvon-Durocher et al., 2015a), by first passing water through a 45 μm and then a 20 μm filter, followed by serial dilution and streaking the isolate on to agar-filled pipette tips turned toward a light source (enabling identification and isolation of motile autotrophs). Organisms putatively identified as *Chlamydomonas* were then grown on agar plates infused with Bold's Basal Medium (BBM). Colonies were then picked under a microscope, transferred back into liquid culture (autoclaved, filtered water taken from rainwater holding tanks at the mesocosm experiment supplemented with BBM at 1/3 the standard concentration) and kept at 18°C (the average daytime temperature across treatments at the time of sampling) for 2 weeks in semi-continuous batch-culture. This yielded concentrations of ${\text{NO}}_{3}^{-}$ at 1,000 μmol L^−1^ and ${\text{PO}}_{4}^{3+}$ at 330 μmol L^−1^. The rationale here was to grow the isolates under nutrient replete conditions (concentrations of N and P that were much larger than observed in the mesocosms) so (i) rates of growth and biomass yields were sufficient to quantify physiological traits, and (ii) the observed cellular stoichiometry would not depend on the availability of nutrients in the medium. Taxonomy of *Chlamydomonas* was confirmed by microscopy and using PCR followed by Sanger sequencing within the 18S sequence using a set of primers with forward sequence GAAGTCGTAACAAGGTTTCC and reverse sequence TCCTGGTTAGTTTCTTTTCC. Positive controls were run using p23 primers amplifying in the RuBisCO region, with forward GGACAGAAAGACCCTATGAA and reverse TYAGCCTGTTATCCCTAGAG. This yielded 18 out of the 20 isolates with an at least 99% BLAST match for *C. reinhardtii*, 8 from heated and 10 from ambient mesocosms. These were used throughout the experiments and the other 2 samples were discarded.

To determine intracellular C, N, and P content, *Chlamydomonas* cultures were grown to exponential phase and the cells counted. A total of 200 mL were spun down at 2,500 r.p.m, the supernatant decanted and the samples frozen immediately in liquid nitrogen until further analysis. Samples for CN content were freeze-dried, weighed out into tin capsules, and analyzed for C and N content using a Sercon 20-22 IRMS. Cell P content was determined via a colorimetric reaction on a Seal Analytics AA3 flow analyser. Pellets were washed in 0.17 M Na_2_SO_4_, transferred to scintillation vials and re-suspended in 4 ml 0.017 M MnSO_4_. The samples were transferred to an autoclave (1 h, 121°C), shaken vigorously and centrifuged at 2,500 r.p.m for 30 min, the pellet discarded, and the supernatant brought to 10 ml with MillliQ purified water. The samples were immediately analyzed on the AA3 using the colorimetric molybdate/antimony method after (Murphy and Riley, 1958).

### Dissolved inorganic nutrients

Water samples for measuring dissolved inorganic nutrient concentrations were collected from mid-depth in each mesocosm at 9 a.m. on each sampling occasion. Samples were filtered (Whatmann GF/F) and stored frozen at −20°C for subsequent determination of ${\text{NO}}_{3}^{-}$, ${\text{NO}}_{2}^{-}$, ${\text{NH}}_{4}^{+}$, and soluble reactive phosphorous (SRP) using a segmented flow auto-analyser (Skalar, San++, Breda, Netherlands) and the methods of Kirkwood (1996).

### Statistical analyses

Seasonal changes in biotic and abiotic variables often exhibit highly non-linear patterns of change, particularly in temperate regions. We therefore used generalized additive mixed effects models (GAMMs) to characterize the seasonal trends and overall treatment effects on temperature, light intensity, GPP, dissolved inorganic nutrients, particulate organic nutrients, and seston stoichiometry. GAMMs do not prescribe any particular functional form for the trend; rather its shape is estimated from the data using penalized regression. GAMMs further account for hierarchical data structures (Zuur et al., 2009). For example, our experimental design yielded replicate seasonal responses for each variable in each treatment. This hierarchical structure meant that measurements were non-independent—e.g., measurements from the same pond will be autocorrelated. We account for this by treating replicate pond as a random effect on the intercept of the model, which models deviations among ponds from the fixed effects as normally distributed with a mean of zero. The full models were specified as follows

(1) $$\begin{array}{l} y_{pt}=\beta +\alpha_{p}+{\text{Treat}}_{t}+f_{\text{Treat}}\left({\text{DOY}}_{t}\right)+\varepsilon_{pt}\\ \varepsilon_{pt}=N\left(0,\sigma^{2}\right)\\ \alpha_{p}=N\left(0,\sigma^{2}\right) \end{array}$$

where *y*_*pt*_ is the response variable in pond, *p*, and time, *t*, β is the intercept, which characterizes the median value of the response variable, “Treat” captures differences in the intercept between treatments (e.g., “warmed” or “ambient”), α_*p*_ is a random effect that characterizes deviations among replicate ponds from the intercept, which we assume are normally distributed with a mean of zero and a variance, σ^2^. The seasonal smooth function, *f*_Treat_(DOY_*t*_), uses a cubic regression spline to model the seasonal trend in the response variable *y*, which is allowed to vary between warmed and ambient treatments. The model residuals, ε_*pt*_, are assumed to be drawn from a normal distribution with a mean of zero and a variance, σ^2^. Model selection entailed fitting a range of models to the data, starting with the full model and then a series of reduced models with interaction terms (e.g., different seasonal smooth functions for each treatment) and main effects removed to test hypotheses about the potential differences in seasonal changes in the response variables among treatments. For multi-model selection we computed small sample-size corrected AIC scores (AICc) and then compared between models by calculating delta AICc values and AIC weights using the “MuMIn” package. When candidate models deviated from the most parsimonious model (that with the lowest AICc score) by less than two AICc units, parameters were averaged across those candidate models using the “model.avg” function in the “MuMIn” package. The relative importance of the fixed factors in the averaged model was determined using the sum of their relative weights. GAMMs were fitted to the data using the “gamm4” package and were conducted in R (v.3.23).

To assess the relative importance of putative abiotic drivers in shaping seasonal variation in seston stoichiometry, we fitted each stoichiometric ratio to the seasonal changes in temperature, light intensity, dissolved inorganic nitrogen (DIN), and SRP in a multiple regression mixed effects model using the “lme” function in the “nlme” package for R.

(2) $$\begin{array}{l} \ln(R_{ip})=\beta_{0}+\alpha_{p}+\beta_{1}T_{ip}+\beta_{2}\ln\left(I_{ip}\right)+\beta_{3}\ln\left(DIN_{ip}\right)\\ \;+\beta_{4}\ln\left(SRP_{ip}\right)+\varepsilon_{ip}\\ \;\varepsilon_{ip}=N\left(0,\sigma^{2}\right)\\ \;\alpha_{p}=N(0,\sigma^{2}) \end{array}$$

where ln(*R*_*ip*_) is the natural logarithm of the *i*th observation of the stoichiometric ratio in pond *p*, β_0_ is the intercept and α_*p*_ is a random effect that characterizes deviations among replicate ponds from overall the intercept, which we assume are normally distributed with a mean of zero and a variance, σ^2^. The slope coefficients, β_1…*n*_ characterize the response of ln(*R*_*ip*_) to the to the various predictor variables. The model residuals, ε_*ip*_, are assumed to be drawn from a normal distribution with a mean of zero and a variance, σ^2^. We natural logarithm transformed the stoichiometric ratios, light intensity, DIN and SRP to linearize all relationships and ensure data were normally distributed prior to statistical analyses. We tested for multi-colinearity by calculating the variance inflation factors (VIF) for each predictor. In each case VIFs were <2.5 indicating that multi-colinearity was low. As for the GAMM analyses, model selection entailed fitting a range of models to the data, starting with the full model (Equation 2) and then a series of reduced models with predictors removed to test hypotheses about the dominant abiotic drivers of seasonal changes in the stoichiometric ratios. Model selection and model averaging was conducted in the same way as described above for the GAMM analyses.

Variation in the taxonomic composition of the phytoplankton communities between treatments and among sampling months was indexed as the “score” for each mesocosm along the first axis of a non-metric multidimensional scaling (NMDS) ordination. NMDS ordination was conducted on each sampling month using the “metaMDS” function in the “vegan” package in R based on a Bray–Curtis dissimilarity matrix derived from log_10_ (X+1)-transformed total abundances of the taxa in each mesocosm. NMDS projected this matrix into a new coordinate space with a small number of dimensions (in this case, 10) while preserving the original Bray–Curtis dissimilarities among samples to the extent possible. Orthogonal rotation was applied to the axes in this new coordinate space so as to maximize the variance in “scores” among samples along the first NMDS axis. Thus, samples with more similar scores along the first NMDS axis are more similar to each other with respect to the dominant gradient in taxonomic composition. We used Permutational Multivariate Analysis of Variance (PERMANOVA) to test whether Bray–Curtis dissimilarities between treatments, months and their interaction were significant.

Because we were interested in assessing the relative importance of species sorting in shaping the seasonal variation and effects of experimental warming on seston stoichiometry, we used the methods described in Baselga and Orme (2012) to partition beta-diversity into its turnover and nested components. We quantify beta-diversity (e.g., spatial or temporal differences in taxonomic composition based on presence-absence data) using the Sorensen's Index (β_sor_), which can be partitioned into components attributable to species turnover (e.g., spatial or temporal replacement of species, β_turn_) and nestedness (e.g., where different sites or time points have species compositions that are nested subsets, β_nes_).

(3) $$\begin{array}{l} \beta_{\text{sor}}=\beta_{\text{turn}}+\beta_{\text{nes}}\equiv \frac{b}{b+a}+\left(\frac{c-b}{2a+b+c}\right)\left(\frac{a}{b+a}\right) \end{array}$$

where *a* is the number of species shared between two sites (or distinct time points), *b* is the number species unique to the poorest site and *c* is the number of species unique to the richest site. We then estimated the fraction of total beta-diversity attributable to turnover (e.g., spatial or temporal replacement of species) as β_frac_ = β_turn_/β_sor_. Using these metrics, we calculated two forms of beta-diversity. First, for each sampling month we estimated spatial beta-diversity as pond-to-pond differences in taxonomic composition, partitioning out variation among ambient, heated and ambient vs. heated ponds. Second, we estimated temporal beta-diversity for each replicate pond by assessing variation in taxonomic composition among sampling months. For both spatial and temporal beta-diversity we calculated β_frac_ = β_turn_/β_sor_ to assess the relative importance of taxonomic turnover between the warmed and ambient treatments as well as over seasonal variation within mesocosms.

## Results

### Seasonal variation and treatment effects on abiotic variables

**Figure 1:**
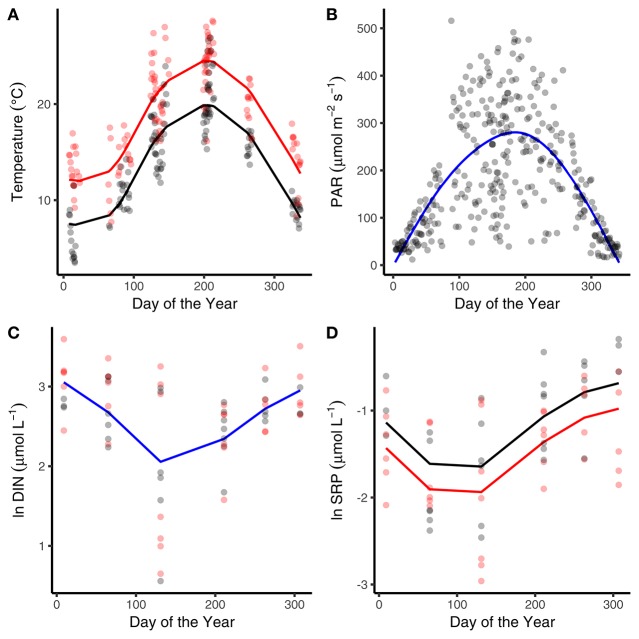
Seasonal variation in abiotic variables. Seasonal changes and treatment effects on **(A)** average daily temperature, **(B)** average daily light intensity, **(C)** dissolved inorganic nitrogen, and **(D)** soluble reactive phosphorous (SRP). Black denotes ambient treatments, red indicates warmed treatments. Fitted lines are from the fixed effects of the best fitting mixed effects models. Where red and black fitted lines are present warmed and ambient treatments differed in either the median value and/or the seasonality of the response variable. Where the fitted line is blue a single function provided the best fit to the data from both treatments.

The abiotic variables measured in the mesocosms showed characteristic seasonality, with temperature and surface water light intensity reaching maxima in July and minima in January (Figures 1A,B). The heated mesocosms were, on average, 4.1°C (±0.7°C) warmer than their ambient counterparts over the entire year (Figure 1A). The seasonality of DIN and SRP reflected drawdown in the spring and early summer, followed by regeneration through autumn and winter (Figures 1C,D). Median SRP levels over the year were lower in the warmed treatments (Figure 1D; Table 1).

**Table 1:** Multi-model selection on generalized additive mixed effects models fitted to the seasonal data.

**Model**	***Df***	**logLik**	**AICc**	**ΔAICc**	**Weight**
**TEMPERATURE**					
**T1 = treat + s(DOY)**	**6.00**	−**156.54**	**326.54**	**0.00**	**1.00**
T0 = treat + s(DOY, by = treat)	8.00	−160.86	340.29	13.76	0.00
T3 = s(DOY)	5.00	−165.54	342.10	15.56	0.00
T2 = s(DOY, by = treat)	7.00	−169.84	355.65	29.11	0.00
**DISSOLVED INORGANIC NITROGEN**					
**DIN3 = s(DOY)**	**5.00**	−**54.06**	**119.13**	**0.00**	**0.74**
DIN1 = treat + s(DOY)	6.00	−53.95	121.34	2.21	0.25
DIN2 = s(DOY, by = treat)	7.00	−55.75	127.46	8.33	0.01
DIN0 = treat + s(DOY, by = treat)	8.00	−55.59	129.74	10.61	0.00
**SRP**					
**SRP1 = treat + s(DOY)**	**6.00**	−**51.69**	**116.82**	**0.00**	**0.73**
SRP3 = s(DOY)	5.00	−54.17	119.35	2.52	0.21
SRP0 = treat + s(DOY, by = treat)	8.00	−51.89	122.36	5.53	0.05
SRP2 = s(DOY, by = treat)	7.00	−54.47	124.90	8.08	0.01
**GROSS PRIMARY PRODUCTION**					
**GPP1 = treat + s(DOY)**	**6.00**	−**54.11**	**121.66**	**0.00**	**0.77**
GPP3 = s(DOY)	5.00	−56.64	124.31	2.64	0.21
GPP0 = treat + s(DOY, by = treat)	8.00	−55.29	129.14	7.48	0.02
GPP2 = s(DOY, by = treat)	7.00	−57.46	130.88	9.21	0.01
**PARTICULATE CARBON**					
**PC3 = s(DOY)**	**5.00**	−**56.31**	**123.63**	**0.00**	**0.89**
PC1 = treat + s(DOY)	6.00	−57.27	128.00	4.36	0.10
PC2 = s(DOY, by = treat)	7.00	−58.46	132.89	9.26	0.01
PC0 = treat + s(DOY, by = treat)	8.00	−59.36	137.28	13.65	0.00
**PARTICULATE NITROGEN**					
**PN3 = s(DOY)**	**5.00**	−**68.19**	**147.40**	**0.00**	**0.81**
PN1 = treat + s(DOY)	6.00	−68.90	151.24	3.84	0.12
PN2 = s(DOY, by = treat)	7.00	−68.28	152.53	5.13	0.06
PN0 = treat + s(DOY, by = treat)	8.00	−68.99	156.55	9.15	0.01
**PARTICULATE PHOSPHOROUS**					
**PP3 = s(DOY)**	**5.00**	−**51.52**	**114.06**	**0.00**	**0.54**
**PP1 = treat + s(DOY)**	**6.00**	−**50.81**	**115.06**	**1.01**	**0.33**
PP2 = s(DOY, by = treat)	7.00	−50.82	117.61	3.56	0.09
PP0 = treat + s(DOY, by = treat)	8.00	−50.32	119.20	5.15	0.04
**C:N RATIO**					
**CN3 = s(DOY)**	**5.00**	−**61.44**	**133.91**	**0.00**	**0.74**
CN1 = treat + s(DOY)	6.00	−61.32	136.09	2.18	0.25
CN2 = s(DOY, by = treat)	7.00	−63.25	142.46	8.55	0.01
CN0 = treat + s(DOY, by = treat)	8.00	−63.12	144.82	10.91	0.00
**C:P RATIO**					
**CP1 = treat + s(DOY)**	**6.00**	−**70.76**	**154.96**	**0.00**	**0.63**
**CP3 = s(DOY)**	**5.00**	−**72.51**	**156.03**	**1.08**	**0.37**
CP0 = treat + s(DOY, by = treat)	8.00	−73.53	165.63	10.68	0.00
CP2 = s(DOY, by = treat)	7.00	−75.33	166.63	11.67	0.00
**N:P RATIO**					
**NP3 = s(DOY)**	**5.00**	−**67.60**	**146.22**	**0.00**	**0.54**
**NP1 = treat + s(DOY)**	**6.00**	−**66.58**	**146.61**	**0.39**	**0.44**
NP2 = s(DOY, by = treat)	7.00	−68.78	153.52	7.31	0.01
NP0 = treat + s(DOY, by = treat)	8.00	−67.78	154.14	7.92	0.01

*A range of models testing hypotheses on the effects of the warming treatment (“treat”) were fitted to the seasonal data; “treat” assess differences in median values warmed and ambient treatments, while comparisons between s(DOY) and s(DOY, by = treat) assess whether the shape of the seasonality differs among treatments. Models were compared via the small sample size corrected Akaike Information Criterion (AICc), delta AICc is the difference in AICc score relative to the model with the lowest value (most parsimonious model) and AICc Weight (Wt) is the relative support for the model. The best fitting models were selected as those returning the lowest AICc score and the highest AICc weight and are highlighted in bold. Where two models differed in <2 AICc units we averaged the coefficients between those models*.

### Seasonal variation in primary production, particulate nutrients and seston stoichiometry

**Figure 2:**
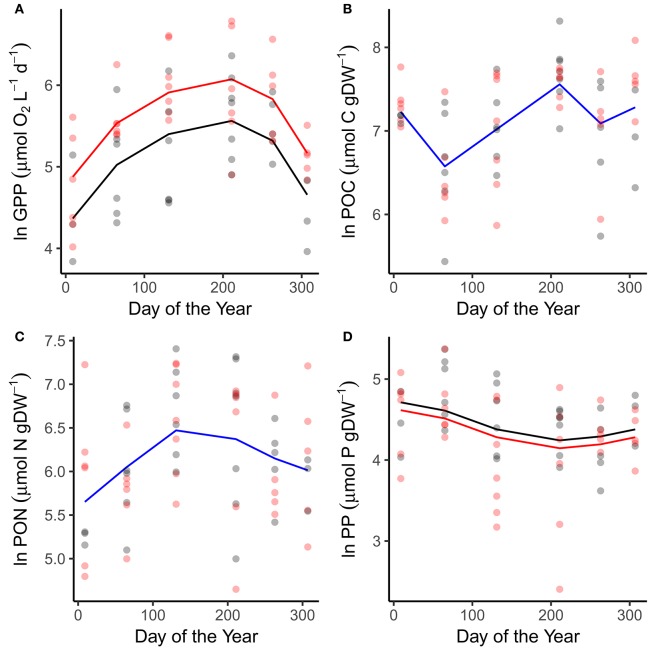
Seasonal variation in primary production and particulate nutrients. Seasonal changes and treatment effects on **(A)** gross primary production, **(B)** particulate organic carbon, **(C)** particulate organic nitrogen, and **(D)** particulate organic phosphorous. Fitted lines are from the fixed effects of the best fitting mixed effects models. Where red and black fitted lines are present warmed and ambient treatments differed in either the median value and/or the seasonality of the response variable. Where the fitted line is blue a single function provided the best fit to the data from both treatments.

Patterns in GPP and phytoplankton nutrient content reflected variation in the abiotic variables. GPP peaked in July in both the warmed and ambient treatments when temperatures and light levels were maximal (Figure 2A; Table 1). Rates of GPP were also elevated in the warmed treatments across all sampling occasions (Figure 2A; Table 1). Seston carbon content peaked in July, in alignment with the maximal rates of GPP, but exhibited no difference between the warmed and ambient treatments (Figure 2B; Table 1). The nitrogen and phosphorous content of the phytoplankton peaked in May and March respectively, in line with the peaks in DIN and SRP drawdown (Figures 2C,D; Table 1). Seston phosphorous content was lower on average in the warmed treatments (Figure 2D; Table 1).

**Figure 3:**
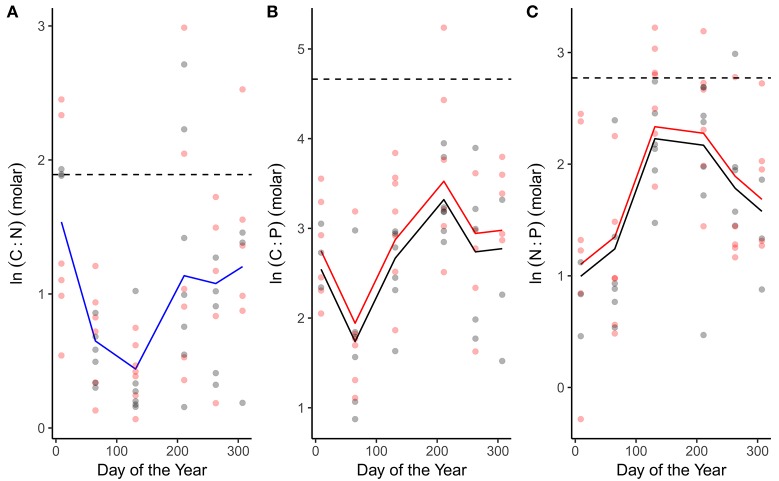
Seasonal variation in phytoplankton stoichiometry. Seasonal changes and treatment effects on **(A)** the C:N ratio, **(B)** C:P ratio, and **(C)** N:P ratio. Fitted lines are from the fixed effects of the best fitting mixed effects models. Where red and black fitted lines are present warmed and ambient treatments differed in either the median value and/or the seasonality of the response variable. Where the fitted line is blue a single function provided the best fit to the data from both treatments. Dashed lines indicate Redfield ratios.

C:N, C:P and N:P ratios all exhibited seasonal variation (Figure 3; Table 1). C:N ratios were highest in winter and declined through spring and summer (Figure 3A; Table 1). C:P and N:P ratios exhibited the opposite seasonal trends, peaking in the spring and summer and declining through autumn and winter (Figures 3B,C; Table 1). In line with our predictions, both C:P and N:P ratios were elevated in the warmed treatments (Figures 3B,C; Table 1) while the C:N ratio was consistent between treatments (Figure 3A; Table 1).

### Abiotic drivers of seston stoichiometry

**Figure 4:**
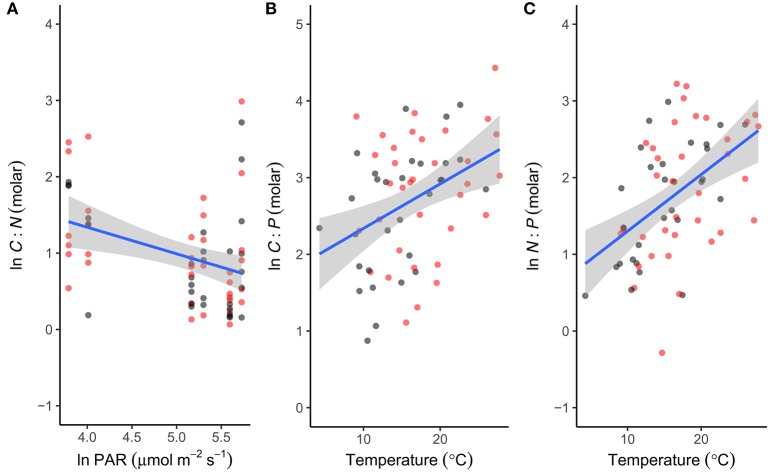
Abiotic drivers of phytoplankton stoichiometry. Correlations between seasonal variation in **(A)** light intensity (PAR) and the C:N ratio, **(B)** temperature and the C:P ratio and **(C)** temperature and the N:P ratio. For each stoichiometric ratio the predictor with the highest summed weight is plotted (see Table 2 for statistics). Fitted lines are from the fixed effects of the best fitting mixed effects models. Black denotes ambient treatments, red indicates warmed treatments.

To investigate the factors shaping the seasonal variation in seston stoichiometry we fitted the data for each elemental ratio to the measured abiotic drivers (temperature, light, DIN, and SRP) using multiple regression in a mixed effects modeling framework. The best fitting model for the C:N ratio included temperature, light, and SRP as predictors (Table 2). C:N ratios were negatively related to light intensity and positively correlated with temperature and SRP (Figure 4A). Temperature, light, DIN, and SRP were all retained as predictors of the C:P ratio in the best fitting model (Table 2), though support for the inclusion of SRP and DIN were weak (i.e., they had low summed weights after model averaging, see Table 2). The C:P ratio was positively correlated with temperature, and negatively related to light (Figure 4B). For the N:P ratio, temperature, light and DIN were all retained in the most parsimonious model (Table 2). The N:P ratio was positively correlated with temperature and light, and negatively related to DIN (Figure 4C). Consistent with our hypotheses, temperature was an important predictor of all the stoichiometric ratios and was the most important predictor for the C:P and N:P ratio (i.e., it had the highest summed weight after model averaging; see Table 2).

**Table 2:** Model selection on multiple regression mixed effects models fitted to investigate abiotic drivers of seston stoichiometry.

**Model**	***Df***	**logLik**	**AICc**	**ΔAICc**	**Weight**
**C:N RATIO**					
PAR+SRP	5.00	−63.43	137.88	0.00	0.27
PAR+ SRP +Temp	6.00	−62.28	138.00	0.12	0.26
PAR+Temp	5.00	−64.01	139.03	1.15	0.15
PAR	4.00	−65.32	139.30	1.42	0.13
	**PAR**	**SRP**	**Temp**		
〈β〉	−0.39	0.24	0.03		
SW	1.00	0.65	0.50		
**C:P RATIO**					
PAR+Temp	5.00	−75.67	162.36	0.00	0.22
PAR+ SRP +Temp	6.00	−74.82	163.09	0.73	0.15
DIN+PAR+Temp	6.00	−74.83	163.11	0.75	0.15
DIN+PAR+ SRP +Temp	7.00	−73.58	163.13	0.77	0.15
SRP +Temp	5.00	−76.16	163.33	0.97	0.14
Temp	4.00	−77.39	163.45	1.09	0.13
	**PAR**	**Temp**	**SRP**	**DIN**	
〈β〉	−0.38	0.08	0.11	−0.08	
SW	0.72	1.00	0.47	0.32	
**N:P RATIO**					
Temp	4.00	−68.89	146.44	0.00	0.30
DIN+Temp	5.00	−67.82	146.66	0.22	0.27
PAR+Temp	5.00	−68.35	147.71	1.27	0.16
	**Temp**	**DIN**	**PAR**		
〈β〉	0.07	−0.07	0.04		
SW	1.00	0.37	0.22		

*Models including all possible combinations of light (PAR) temperature (Temp), dissolved inorganic nitrogen (DIN), and soluble reactive phosphorous (SRP) as predictors of the stoichiometric rations were compared via the small sample size corrected Akaike Information Criterion (AICc), where delta AICc is the difference in AICc score relative to the model with the lowest value (most parsimonious model) and AICc Weight (Wt) is the relative support for the model. The best fitting models were selected as those returning the lowest AICc score and the highest AICc weight. Where models differed by <2 AICc units we averaged the coefficients between those models. All models with delta AICc < 2 are given in the table above with model averaged coefficients, 〈β〉 and the relative importance of each parameter, given by SW, which ranges from 0 (no models in the final set retain the parameter) to 1 (all models in the final set retain the parameter)*.

### Seasonal variation and treatment effects on phytoplankton community composition

**Figure 5:**
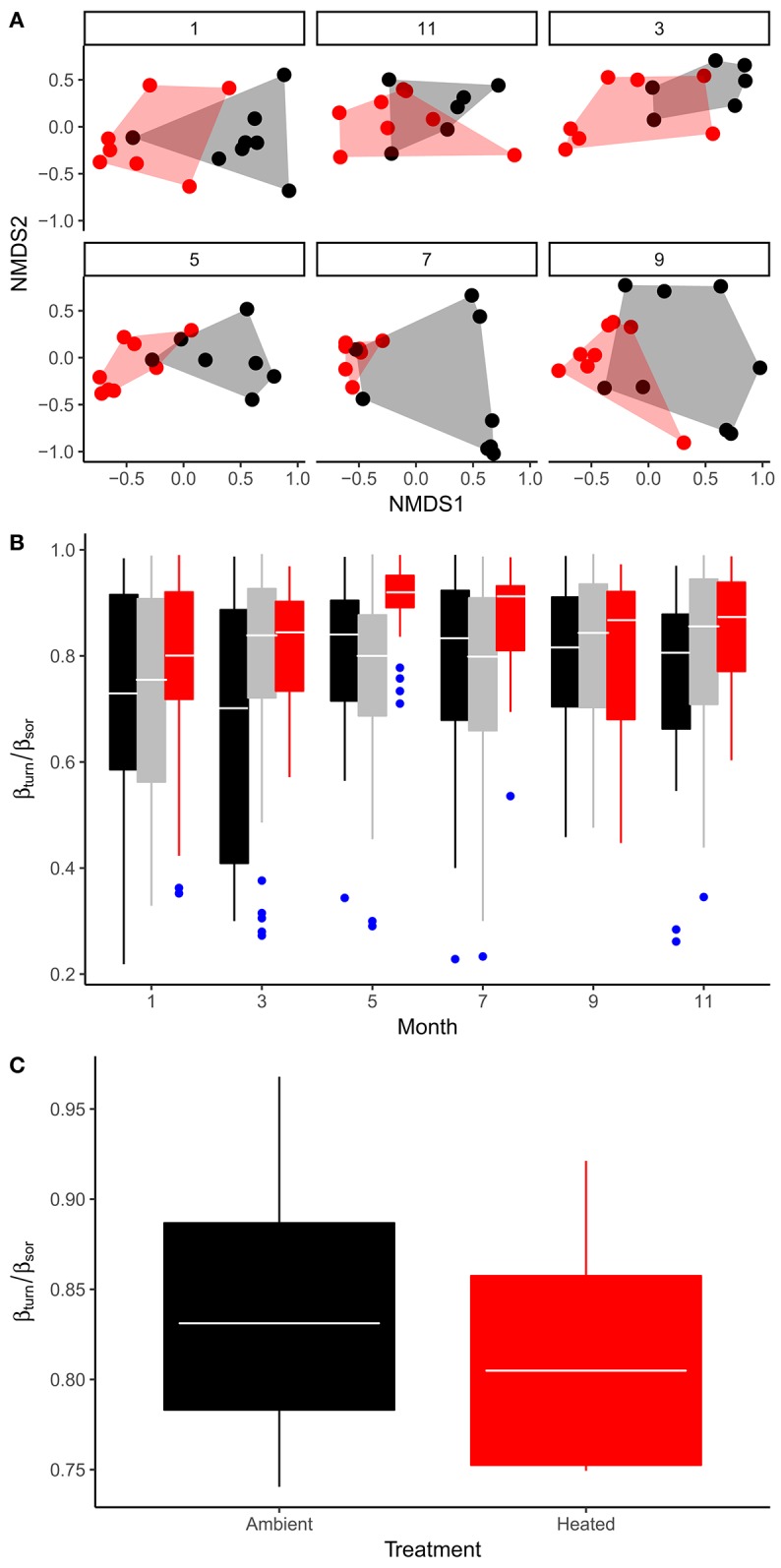
Seasonal variation and treatment effects on phytoplankton community structure. **(A)** Non-metric multidimensional scaling (NMDS) of phytoplankton community composition comparing treatment effects across sampling months (1 = Jan, 3 = Mar, 5 = May, 7 = Jul, 9 = Sep, 11 = Nov). **(B)** Seasonal variation in the fraction of total beta-diversity among ponds that is attributable to taxonomic turnover (β_turn_/β_sor_), here black boxes encompass variation in β_turn_/β_sor_ among ambient replicates, red show variation between warmed replicates and gray denotes variation in beta-diversity derived from comparisons among warmed vs. ambient replicates. **(C)** Treatment effects on β_turn_/β_sor_ derived from temporal comparisons of community composition among sampling months within each mesocosm. Tops and bottoms of boxes in box-whisker plots correspond to the 25th and 75th percentiles, horizontal white lines correspond to medians, whisker extents correspond to 1.5 × the interquartile range.

The effects of seasonal variation in temperature and the other abiotic variables, as well as the effect of experimental warming on the bulk stoichiometry of the phytoplankton, will be mediated by a combination of factors, including (i) physiological acclimation of cellular stoichiometry within species; (ii) evolutionary change in response to the long-term warming treatment; (iii) environmentally driven species sorting; both in response to seasonal changes in temperature within ponds as well as the long-term temperature differential maintained between the warmed and ambient treatments. To assess the potential importance of species sorting and temperature-dependent variation in phytoplankton community composition on seston stoichiometry we quantified the taxonomic composition and relative abundance of the phytoplankton communities in the mesocosms on 6 sampling occasions over an annual cycle. We found significant variation in phytoplankton community composition both among treatments [Figure 5A; PERMANOVA, *F*_(1, 89)_ = 9.2; *P* < 0.001] as well as across sampling occasions [Figure 5A; PERMANOVA, *F*_(5, 89)_ = 2.3; *P* < 0.001], based on Bray–Curtis dissimilarities. To determine the extent to which pond-to-pond and month-to-month differences in species composition (e.g., spatial and temporal beta-diversity respectively) were driven by species replacements vs. species losses, we quantified the relative proportion of total beta-diversity attributable to taxonomic turnover and nestedness (Baselga and Orme, 2012). On all sampling months, spatial beta-diversity was driven primarily by turnover in species composition among ponds, which was consistent when comparing across treatments—e.g., on average, beta-diversity attributable to taxonomic turnover was responsible for 78% of total beta-diversity (Figure 5B). Similarly, temporal beta-diversity within ponds also predominantly reflected turnover in composition rather than nestedness (Figure 5C). These analyses demonstrate that ~80% of the variation in taxonomic composition of the phytoplankton communities both in response to seasonal changes in temperature and the effects of experimental warming were driven by replacements of species.

### Effects of thermal adaptation on phytoplankton stoichiometry

**Figure 6:**
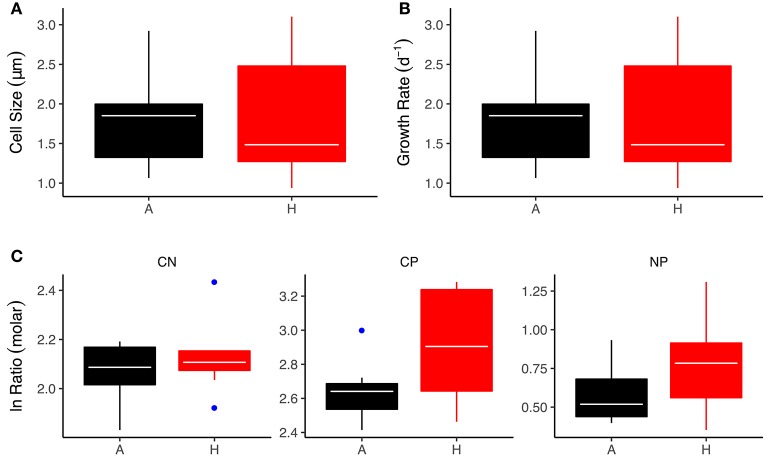
Effects of experimental warming on physiological and stoichiometric traits of *C. reinhardtii* isolates. **(A)** Cell size at 18°C, **(B)** Growth rate at 18°C, **(C)** Stoichiometric ratios. Black indicates the ambient treatments and red the warmed. Tops and bottoms of boxes in box-whisker plots correspond to the 25th and 75th percentiles, horizontal white lines correspond to medians, whisker extents correspond to 1.5 × the interquartile range and blue points are outliers.

The green alga, *C. reinhardtii*, was one of the most abundant (top 10% of all species across the meta-community) and widely distributed taxa across the meta-community, with established populations in all warmed and ambient mesocosms. To investigate whether thermal adaptation resulted in changes in cellular stoichiometry that might contribute to the effects of warming on bulk seston stoichiometry we measured C:N:P ratios in strains of *C. reinhardtii* isolated from the warmed and ambient treatments. Differences in the stoichiometric ratios between the warmed and ambient isolates closely matched the treatment effects on bulk seston stoichiometry. The C:N ratios were not significantly different between warmed and ambient isolates [Figure 6A; ANOVA Type-III; *F*_(1, 16)_ = 2.52, *P* = 0.13]. The C:P ratio was significantly elevated in the warmed isolates [Figure 6B; ANOVA Type-III; *F*_(1, 16)_ = 6.40, *P* = 0.02], in line with the higher seston C:P ratios observed in the warmed treatments and the positive correlation between seasonal variation in temperature and bulk phytoplankton stoichiometry (Figures 3, 4). The N:P ratio was marginally, but not significantly, elevated in the warmed isolates [Figure 6C; ANOVA Type-III; *F*_(1, 16)_ = 2.49, *P* = 0.13]. This weaker effect of warming on the N:P ratio was also consistent with the smaller effect size of warming on the seston N:P ratio (Figure 3) and the weaker seasonal temperature dependence of phytoplankton N:P (Figure 4), relative to the effects of warming and temperature on the C:P ratio.

## Discussion

Understanding how ecosystem-level properties, like the bulk stoichiometry of plankton, are shaped by selection on trait variation within and across species is key to improving predictions of global change on biogeochemical cycles (Hagstrom and Levin, 2017). Central to this issue is a grasp of the relative importance of rapid evolution (i.e., changes in the frequency of traits within species populations) and species sorting (i.e., changes in the frequency of traits attributable different species) in shaping how ecosystem-level properties respond to environmental change (Lomas et al., 2014). We tackled this issue by assessing the extent to which the effects of long-term experimental warming and seasonal changes in temperature on the C:N:P stoichiometry of phytoplankton in pond mesocosms reflected temperature-dependent changes in the composition of the communities vs. evolutionary shifts in stoichiometric traits within component species. We found that warming resulted in substantial shifts in phytoplankton community composition, consistent with temperature-driven species sorting. Furthermore, isolates of *C. reinhardtii* from warmed mesocosms had higher C:P and N:P ratios than their ambient counterparts, with shifts that were comparable in direction and magnitude to the effects of warming on seston stoichiometry. These analyses suggest that both species sorting and rapid local adaptation could have contributed to the effects of warming on bulk phytoplankton stoichiometry.

We found higher average C:P and N:P ratios driven by lower particulate P content in the seston from the warmed mesocosms, as well as positive correlations between seasonal changes in temperature and C:P and N:P ratios in both the warmed and ambient treatments. These findings add to a growing body of evidence demonstrating positive covariance between temperature and C:P and N:P ratios in aquatic and terrestrial autotrophs (Reich and Oleksyn, 2004; Martiny et al., 2013; Toseland et al., 2013; Yvon-Durocher et al., 2015b). However, a critical question concerning the mechanisms underlying these patterns is the extent to which they are driven by evolutionary flexibility in stoichiometric traits within species vs. temperature driven turnover in taxonomic composition along thermal gradients.

To investigate the role of species sorting we quantified the extent to which differences in phytoplankton communities between the warmed and ambient treatments as well as across sampling months, reflected turnover in species composition (e.g., replacement of species across space and/or time). We found that ~80% of the variation in phytoplankton composition among treatments and sampling months could be attributed to taxonomic replacements (e.g., turnover as opposed to nestedness). This result demonstrates that the composition of the phytoplankton communities were highly dynamic both in response to seasonal abiotic change and sustained increases in temperature between the treatments. Consequently, this result implies that species sorting and associated changes in community-wide traits could have played an important role in shaping the effects of temperature on bulk stoichiometry, consistent with recent work in marine ecosystems (Irwin et al., 2015; Edwards, 2016; Martiny et al., 2016b). Temperature-driven species sorting could affect community-level bulk stoichiometry in two ways. First, phytoplankton stoichiometry is known to exhibit substantial variation among the major taxonomic clades (Quigg et al., 2003; Finkel et al., 2016). For example, red lineage algae, which include the diatoms, have relatively low N:P ratios, while green lineage algae, which include the chlorophytes, tend have higher N:P ratios (Quigg et al., 2003). Thus, changes in phytoplankton community composition, where taxa with high average C:P and N:P ratios are favored in warmer environments and those with low values are abundant in cooler conditions could conceivably be an important factor shaping the effects of temperature on bulk phytoplankton stoichiometry. Second, it is also possible that C:N:P stoichiometry is phenotypically plastic with respect to temperature change in a consistent way across species (Yvon-Durocher et al., 2015b) and the observed turnover in community composition reflects selection for traits other than stoichiometry. In this case, the composition of the community changes with temperature and the stoichiometry of the constituent species also shift with temperature, not because different taxa have divergent stoichiometry, but because the reaction norm for temperature-driven stoichiometric plasticity is conserved across species. Unfortunately, our data do not allow us to differentiate between these two possibilities, as this would require detailed acclimation experiments to be conducted on a wide range of the species that comprise the phytoplankton communities in the experiment. It is important to note however, these two scenarios are not mutually exclusive and there is evidence for both conserved temperature driven stoichiometric plasticity across species (Yvon-Durocher et al., 2015b) as well as systematic taxonomic variation in average C:N:P ratios (Quigg et al., 2003; Finkel et al., 2016).

To investigate whether rapid evolutionary shifts in stoichiometric traits in response to warming played a role in the effects of warming on phytoplankton bulk stoichiometry we isolated the abundant and cosmopolitan alga *C. reinhardtii*, which was present across both warmed and ambient treatments. We have previously shown, through reciprocal transplants, that isolates of *C. reinhardtii* are locally adapted with respect to the warming treatment, with warmed isolates incuring a reduction in competitive fitness when transplanted to ambient temperatures and ambient isolates having reduced fitness when exposed to warming (Schaum et al., 2017). Our analyses here demonstrate that the warm-adapted isolates had C:P ratios that were 33% higher than their ambient counterparts. This within taxon response was remarkably close to the overall effect size on average bulk seston C:P with ratios that were 38% higher in the warmed treatments. The N:P ratio was marginally, but not significantly elevated in the warm-adapted isolates of *C. reinhardtii*. However, notwithstanding the absence of a significant treatment effect, the effect sizes between the isolates and the bulk community response to warming in the N:P ratio were also very similar, a 21 and 27% increase in response to warming in the isolates and the bulk seston respectively. These results demonstrate that selection on stoichiometric traits within and across species in response to warming are of a similar direction and magnitude, implying that that thermal adaptation also likely contributed the shifts in bulk seston stoichiometry in response to long-term experimental warming.

Our findings of elevated C:P and N:P ratios with increases in temperature, both at the species- and community-levels, are broadly consistent with the “*temperature-dependent physiology*” hypothesis, which predicts that fewer P-rich ribosomes are required at warmer temperatures owing to the increased efficiency of ribosomes at higher temperatures (Woods et al., 2003; Toseland et al., 2013; Yvon-Durocher et al., 2015b). Average C:P and N:P ratios both in the seston and in the isolates of *C. reinhardtii* were however very low (seston C:P = 16.6, seston N:P = 6.3; isolate C:P = 17.2, isolate N:P = 4.2), indicating that luxury uptake and storage of phosphorous may have contributed significantly to the cellular P content. Given the nanomolar concentrations of SRP in the mesocosms, luxury storage of P, which is known to be an adaptation to severe nutrient limitation, is a plausible explanation for the very low C:P and N:P ratios. Nevertheless, growth rates and cell sizes of the *C. reinhardtii* strains were comparable between the warmed and ambient isolates (see Figure 6) indicating that whilst luxury P storage might explain the overall low C:P and N:P ratios it is unlikely to be the underlying driver of the effects of warming.

The low C:P and N:P ratios in the seston appear at odds with the nanomolar concentrations of SRP in the mesocosms and raise the fundamental question of where the algae are sourcing the phosphorous required to sustain such high C:P and N:P ratios. It is notable, that many of the algae that are numerically dominant in both the warmed and the ambient mesocosms are capable of mixotrophic growth (e.g., *C. reinhardtii, Cryptomonas* spp., *Gymnodinium* spp.) and are known to supplement their nutritional requirements with resource uptake via osmotrophy or phagotrophy (Spero and Morée, 1981; Tranvik et al., 1989; Tittel et al., 2005). The fact that seston C:P and N:P ratios are only weakly correlated with seasonal changes in SRP suggests that uptake of P from organic sources (dissolved organic P or P associated with bacterial prey) could be an important component of the phosphorous biogeochemistry of these oligotrophic systems.

Overall our experiments demonstrate a striking coherence between stoichiometric responses to temperature change between the long-term warmed and ambient mesocosms, across seasonal variations in temperature, and between strains of a cosmopolitan alga isolated from the long-term experiment. The consistency in the effects of temperature in driving increases in C:P and N:P stoichiometry both within species as an adaptive response to the long-term warming as well as at the community level via species sorting, implies that temperature driven adjustments in sub-cellular allocation to C, N and P pools that increase C:P and N:P in warmer environments increase fitness and can be reinforced through ecological and evolutionary processes. Our findings demonstrate highly conserved responses of elemental stoichiometry to temperature across multiple spatial, temporal and organizational scales and highlight that profound changes in aquatic biogeochemistry should be expected in a warmer world.

## Author contributions

GYD and MT conceived the study. GYD and CS conducted the experiments. GYD analyzed the data. GYD wrote the manuscript and all authors contributed to revisions.

### Conflict of interest statement

The authors declare that the research was conducted in the absence of any commercial or financial relationships that could be construed as a potential conflict of interest.
